# Gold Mining in the Peruvian Amazon: Global Prices, Deforestation, and Mercury Imports

**DOI:** 10.1371/journal.pone.0018875

**Published:** 2011-04-19

**Authors:** Jennifer J. Swenson, Catherine E. Carter, Jean-Christophe Domec, Cesar I. Delgado

**Affiliations:** 1 Nicholas School of the Environment, Duke University, Durham, North Carolina, United States of America; 2 Ecole Nationale des Ingénieurs des Travaux Agricoles de Bordeaux, Unité Mixte de Recherche Transfert et Cycle des Éléments Minéraux, Gradignan, France; 3 Environmental Affairs Office, CESEL S.A, San Isidro, Lima, Peru; University of Bristol, United Kingdom

## Abstract

Many factors such as poverty, ineffective institutions and environmental regulations may prevent developing countries from managing how natural resources are extracted to meet a strong market demand. Extraction for some resources has reached such proportions that evidence is measurable from space. We present recent evidence of the global demand for a single commodity and the ecosystem destruction resulting from commodity extraction, recorded by satellites for one of the most biodiverse areas of the world. We find that since 2003, recent mining deforestation in Madre de Dios, Peru is increasing nonlinearly alongside a constant annual rate of increase in international gold price (∼18%/yr). We detect that the new pattern of mining deforestation (1915 ha/year, 2006–2009) is outpacing that of nearby settlement deforestation. We show that gold price is linked with exponential increases in Peruvian national mercury imports over time (R^2^ = 0.93, p = 0.04, 2003–2009). Given the past rates of increase we predict that mercury imports may more than double for 2011 (∼500 t/year). Virtually all of Peru's mercury imports are used in artisanal gold mining. Much of the mining increase is unregulated/artisanal in nature, lacking environmental impact analysis or miner education. As a result, large quantities of mercury are being released into the atmosphere, sediments and waterways. Other developing countries endowed with gold deposits are likely experiencing similar environmental destruction in response to recent record high gold prices. The increasing availability of satellite imagery ought to evoke further studies linking economic variables with land use and cover changes on the ground.

## Introduction

World demand for natural resources is increasingly driving local resource extraction and land use [1]. As the global economy becomes more tightly connected, it is increasingly difficult for developing countries to harness the lucrative forces of global demand in the interest of social and environmental sustainability [2]. As a result, developing countries become saddled with an unequal environmental burden relative to developed countries that are importing the raw materials [3].

A current example of a global commodity having such an effect in developing countries is gold. Over the last decade, the price of gold has increased 360% with a constant rate of increase of ∼ 18% per year. The price continues to set new records, rising to over >$1400/oz at the time of this article's publication [4]. As a response, nonindustrial informal gold mining has risen in developing countries along with grave environmental and health consequences [5], [6]. “Informal” refers to artisanal miners that operate illegally without paying taxes or holding permits and/or formal title to their claims [7], [8] and without environmental impact analysis or miner education. Artisanal gold miners are typically the poorest and most marginalized in society [5], [8], [9], and therefore are difficult to target and regulate with education and incentives. Gold mining activity has seen surges in response to global markets in the past in this region [7], but seems to be increasing to new wide-spread levels as a response to record high prices [8], [10], [11].

Major environmental threats caused by gold mining in the developing world include deforestation, acid mine drainage, and air and water pollution from arsenic, cyanide, and mercury contamination [12]. The environmental and health problems caused by mercury are well documented [13], yet its use continues to be an intrinsic component in today's artisanal mining [8], [14]. Artisanal miners are directly exposed to liquid mercury as well as to vapors during gold processing, which releases mercury directly into sediments, waterways and the atmosphere. It is estimated globally, that artisanal mining since 1998 produces 20–30% of global gold production [12] and is responsible for one third (average of ∼1000 t/y) of all mercury released in the environment [14].

While many developing countries have reached environmental accords with large gold mining companies that typically do not use mercury, they continue to struggle in the control and regulation of artisanal mining [12] especially in remote areas. Peru, likely to be the fifth largest gold producer in the world this year [15], does not restrict mercury imports. Peruvian mercury imports have risen 42% (2006 to 2009) to 130 t/yr (Superintendencia Nacional de Aduanas del Perú in [10]). Over 95% of this imported mercury is used directly in artisanal mining (Superintendencia Nacional de Aduanas del Peru in [16]). The ratio of mercury use to resulting gold amalgam is at least 2 to 1 in artisanal mining [8], [12], yet there is no information on mercury use nor its transfer within the country at the department level. Estimates of mercury lost and gold extracted are notoriously hard to acquire for artisanal mining [12].

Peru's Department of Madre de Dios provides us with an example typical of many other “low-governance” areas of the world (*sensu* [17]). Land use here is not well regulated, appears to be determined by local private interests, and is changing rapidly [18]. The area is subject to an increasing poor migrant population and ever-expanding resource extraction [19], [10]. Past land use change in this region has been influenced by roads and urban areas [20] as well as rural credit programs [21], but currently, large continental-scale multi-faceted infrastructure projects are providing new intercontinental access to the region [22]. The Department of Madre de Dios is Peru's third largest producer of gold, and generates 70% of Peru's artisanal gold production (Ministry of Energy and Mines 1998 *in* [16]). The number of informal miners is not known, nor therefore is the percent that have applied for mining permits. However, the Madre de Dios Department has the highest number of unapproved mining permits of the country (1016, as of July 2009, [23]). Acquiring a permit requires an environmental impact report, yet because there is currently little effective enforcement neither of unapproved permits nor of illegal miners [8], [10], [11], there is less incentive to apply for a permit.

Madre de Dios Department occupies Peru's lowland Amazon which is globally recognized as one of the most biologically rich and unique areas on Earth and is proclaimed by Peruvian law (N^o^. 26311.), to be the “Capital of Biodiversity”. This region is part of one of the largest uninterrupted expanses of forest remaining in the Amazon. The western Amazon, in addition to having a high diversity of human indigenous cultures [24], hosts the highest number of mammal [25], avian [26], and amphibian [27] species in the continent, and is one of the most biodiverse areas in the world [28]. The natural and protected areas in the region also provide important economic benefits to the region through nature-based tourism [18].

**Figure 1:**
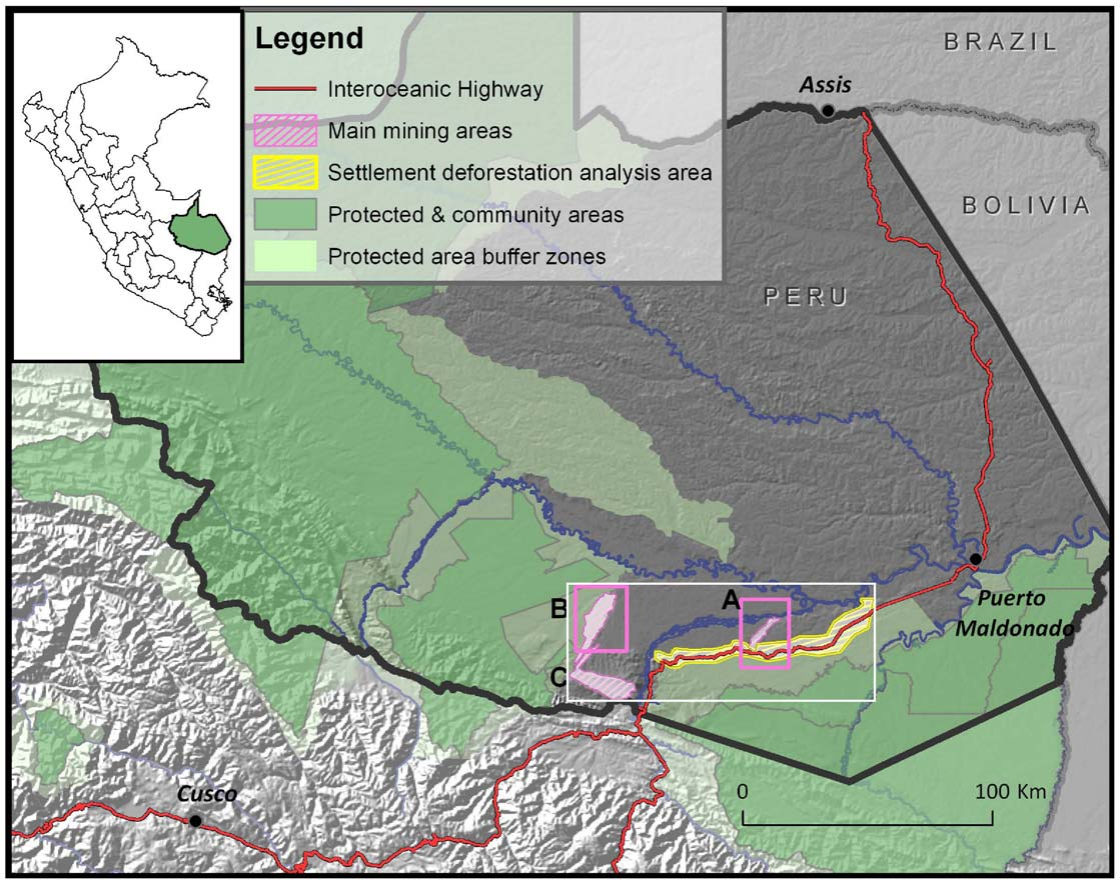
Study area, Department of Madre de Dios in Peru. Mining areas denoted by “A”, for Guacamayo (Figure 2A), “B” Colorado-Puquiri (Figure 2B), and “C”, Huepetuhe. Shown with white box of immediate study area, national protected and community areas and their buffer zones, and topography.

The backdrop of the densely forested Amazon basin offers a unique opportunity to trace the recent expansion of mined area that would be difficult or implausible in sparsely vegetated regions. Recent mining can resemble naturally barren areas such as found in the high Andes and along river sandbars. Here we map and quantify the conversion of forest (2003–2009), specifically for gold mining in the Madre de Dios region in the Amazon Basin, Peru, using satellite images. We then examine the relationship between increases in global gold prices, mined area, and Peruvian mercury imports. For a comparison to area mined, we concurrently monitored nearby “settlement” deforestation, area cleared for human habitation and non industrial activities such as agriculture, along the main transportation artery, the Interoceanic Highway (IOH) that now links Brazil and Peruvian ports (Figure 1).

## Methods

### Study area

**Figure 2:**
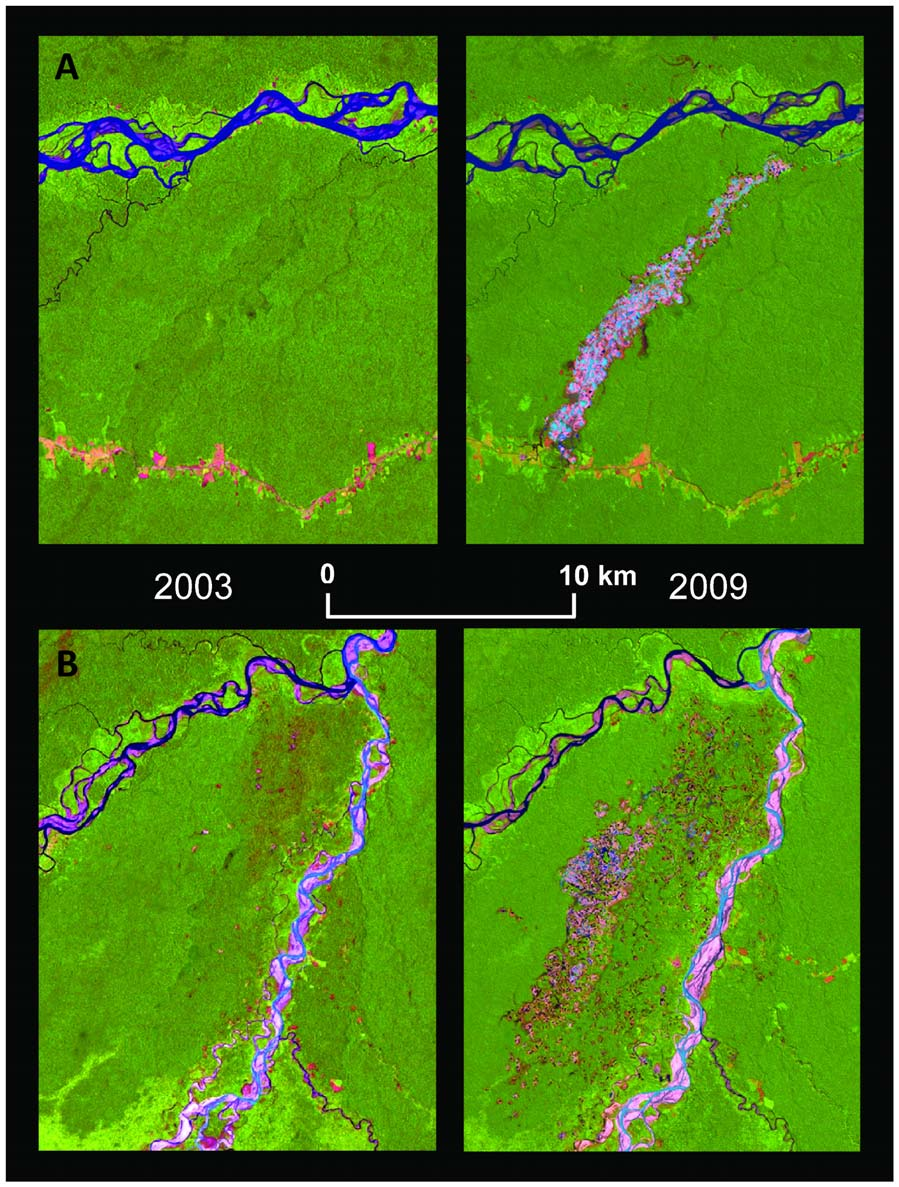
Satellite images of recent mining activity (Landsat TM bands 5, 4, 3); A) Guacamayo (12°51′S, 70°00′W) along the IOH and (B) Colorado-Puquiri (12°44′S, 70°32′W) in the buffer zone of the Amarakaeri Communal Reserve.

The Peruvian department of Madre de Dios in SE Peru covers ∼85,000 km^2^ of primarily Amazon lowland rain forest (Figure 1). There is an extensive network of navigable rivers and an increasing road network that crosses into neighboring Brazil and Bolivia. We focus on the two main incipient mining areas in the Department of Madre de Dios, Peru: Guacamayo [Huacamayo], between the Inambiri River and the IOH (Figure 1, “A”, and Figure 2A), and an area between the Colorado and Puquiri [Pukiri] rivers often referred to as “Delta 1” (Figure 1, “B”, and Figure 2B). A third larger mine, Huepetuhe [Huaypetuhe] (Figure 1, “C”), begun in earnest in the 1980's, continues to produce the most artisanal gold, though has lower rates of recent areal increase since 2003.

### Mapping

We analyzed satellite imagery from 2003 to 2009 to identify mined areas for the two principal mines that have grown substantially. We used Landsat 5 Thematic Mapper (path 3, row 69) satellite imagery [29] for the dates: October 15, 2003, August 4, 2006, and August 28, 2009. We atmospherically and radiometrically corrected the images and found them to be cloud free for our areas of interest.

In this region, mined area provides high contrast with the background forest and has a distinct spectral signature from that of typical settlement deforestation. Mining areas however, may be confused with river features of open water and sandbars. To alleviate this problem we geographically isolated mining areas by hand-digitizing polygons around them that avoided rivers and naturally exposed soil and sand bars. We tested different supervised classification approaches yet elected to isolate geographical areas and use unsupervised classification. We applied an ISODATA classification over each of the discrete areas with 40 classes per site. Classes were separated into forest, edge or secondary forest, and mining by visual interpretation based on the image bands, tasseled-cap indices and the normalized difference vegetation index. Mining included a range of subclasses from water bodies to exposed soil surfaces.

For a general comparison of mined area to other deforestation, we isolated “settlement” deforestation along 100 km of the IOH that included all adjacent deforested areas within 4 km of the highway (not including rivers). We chose this area as it has the highest amount of deforestation within the ∼34,000 km^2^ satellite image. This 100-km transect of the IOH reached from Santa Rosa to Floridabaja before Puerto Maldonado. We applied an ISODATA classification with 60 classes. Some deforestation classes included limited areas of bamboo forest that were present during both years analyzed. A portion of the 2009 map was assessed for accuracy based on available high resolution imagery (see Table S1 for results).

### Data analysis

We used regression to examine relationships among variables. Biweekly global gold prices [4] and mercury imports, monitored by the Superintendencia Nacional de Aduanas del Perú (Peruvian customs) were acquired for 2000 to 2006 [16], and for 2006 to September 2009 [10]; for the last quarter of 2009, imports were calculated as an average based on the three previous quarters of 2009. We graphed increase over time in gold price, deforestation, and mercury imports. For rates of increase we fit curves to the original data and used these to project mercury imports over the near future given the current rate of increase in gold price.

## Results

**Figure 3:**
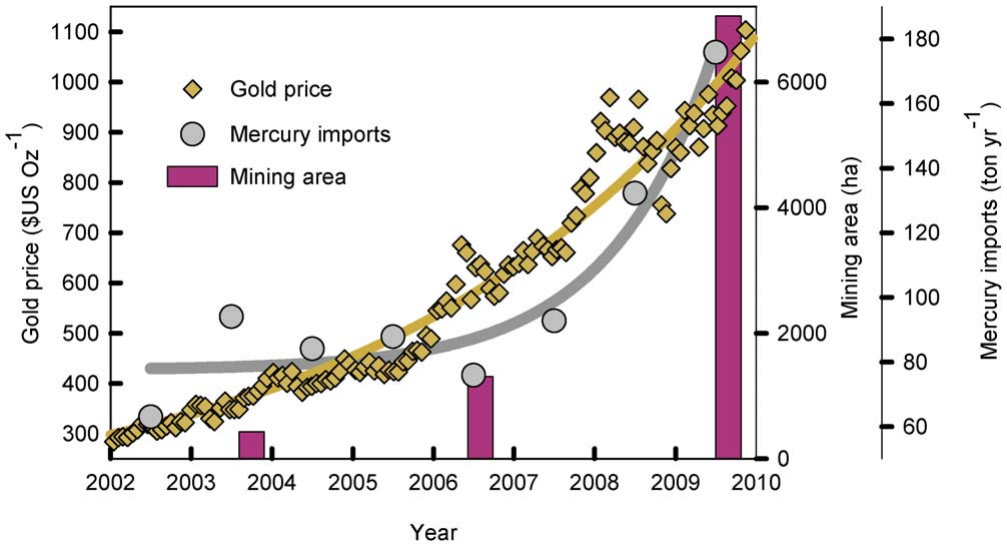
Gold, deforestation, and mercury import increases over time. International biweekly gold prices [4], forest conversion to mining area (Figure 2A and B) and annual mercury imports to Peru (Superintendencia Nacional de Aduanas del Perú [14], [9]). Mercury imports for 2009 were recorded to September and projected for last quarter. Gold price had risen to >$1400/oz at the time of this article's publication [4].

We found that recent mining is converting primary forest at a non-linear rate alongside increasing gold prices (Figures 3 and S1). From 2003 to 2009, mining in the two sites has converted ∼6600 ha of primary tropical forest and wetlands, to vast expanses of ponds and tailings (Figure 2). In conjunction with annual rate of increase in gold prices of ∼18%/yr, forest conversion to mining in these sites increased six-fold from 2003–2006 (292 ha/yr) to 2006–2009 (1915 ha/year). From 2002 to 2009 gold prices were significantly related to mercury imports (R^2^ = 0.93, mercury Imports = 80.77+0.0054 e^(0.0105*^ ^gold price)^ p = 0.04; Figure S1, B). Given the past rates of gold price and mercury imports, we predict future mercury import increases of 64% for 2010 (estimated ∼280 t/yr), which may nearly double by 2011 (based on 18% increase in 2011 gold price; Figure S1, B, Table S2). The three mining sites combined (Guacamayo, Colorado-Puquiri, and Huepetuhe) totaled ∼15,500 ha of mined area as of August 2009 (Table S3). The large mining sites farthest west (Figure 1,”B” and “C”) lie partially inside the buffer zone <7 km from the Amarakaeri Communal Reserve and <70 km from Manu National Park Biosphere Reserve. Numerous smaller sites are scattered throughout riparian and wetland areas within the area which have the potential to grow spontaneously in the future. From our examination of settlement (nonmining) deforestation along 100 km of newly paved pre-existing IOH (within 4 km of the highway, an area of ∼82,250 ha), we found that ∼15.5% of this 4-km zone has been deforested but with a relatively modest annual increase of ∼220 ha/yr (2006–2009, Table S1). Of the immediate area (a 46×120-km rectangle encompassing all study sites), mining in 2009 comprises 2.8%, while settlement deforestation covers 2.3% of the area (Table S3).

## Discussion

Our finding of increasing rates of mining deforestation, together with the continuing increase of annual rate of gold price and an exponential rise in mercury imports, bode poorly for the future of these ecosystems and communities. Recent increases in artisanal mining in developing countries are still largely lacking the aid of technology, regulation, or timely study [5]. Allowing mining to continue without environmental regulation and permitting unrestricted mercury imports has and will continue to result in negative long term environmental and health consequences.

The influx of miners to pristine or sparsely inhabited areas may increase the hunting of native wildlife (e.g. professional hunters stockpile wild game for miners in Nouragues Natural Reserve, French Guyana [30]), disturb indigenous communities (e.g. in Venezuela[31] and Guyana [32]), and fragment once large and pristine forest blocks. Detailed data collected for 54 national parks in 7 Latin American countries indicate that mining is considered a threat in 37% of the parks, 55% of those were located in Peru [33]. Management attempts at controlling renegade mining have ranged from ineffective airborne patrols of French police in French Guyana [30], to border controls for the influx of Brazilian ‘wildcat’ miners (*garampiros*) to Guyana [32]. Because of the miners’ ability to use waterways for transportation, they are capable of invading the far reaches of community and protected areas. The protection of these areas is hindered by the lack of funding, staff and staff training, as well as the difficulty of patrolling such remote extents [34]. Monitoring of illegal mining would be feasible with frequent high resolution (e.g. <10 m) satellite imagery. Unfortunately, images that are consistently collected at this resolution are not freely available currently, and steady monitoring can be costly.

Though increased gold mining is occurring worldwide, our study of the gold-mercury relationship is restricted to the country of Peru. Our methods may slightly underestimate mercury imports for the last quarter of 2009; we have averaged the previous quarters of 2009 to estimate the final quarter. The growth curve fit to our data should be interpreted with caution: our sample size is small, and there are other factors that may likely dampen the predicted growth in mercury imports, such as the depletion of more accessible gold deposits. A more conservative approach to the gold-mercury model could be to project mercury imports with a linear function beginning in 2006, when imports began to climb. Based on 2006–2009, a linear function (Adj. R^2^ = 0.89, p <0.04) projects a 36% increase in mercury imports in 2010 (240 t/yr) and a 68% increase by 2011 (300 t/yr), relative to imports in 2009 (exponential predictions based on 2003–2009, are 64 and 100%, respectively).

Our deforestation measurements focus upon the major mining growth centers within the Madre de Dios department over the past six years—mining areas we can map with certainty. However, there are many scattered small expanding areas of mining across the Department that prove more challenging to detect by satellite because of their similar reflectance to migrating river corridors. Our estimates were designed to focus on these major increases in the region in a timely fashion, whereas future work could strive to accurately map department-wide mining activity, with higher resolution imagery and extensive field validation. We suspect the situation in Madre de Dios is simultaneously occurring in many parts of the developing world. Our mapping methods were designed for surface mining amid a dense forest backdrop, and may not be applicable in different areas such as the arid Andean highlands. Given the data limitations in this region of the world, specifically the lack of reliable estimates of mining rates at the national or department level, lack of data on mercury distribution within the country, and poor estimates of the percentage of legal vs. informal miners, our results trace a substantial increase in mining area by satellite coupled with global economic price increases and mercury imports.

There has been concern about the recent infrastructure improvements such as the IOH in terms of the anticipated rates of forest conversion [35]. However, perhaps of greater concern in this area, is that we find mining deforestation is increasing markedly over time and appears to be outpacing settlement deforestation during these recent years. We characterized settlement deforestation rate (∼220 ha/year) along the nearby IOH for a relative comparison to mining deforestation rate (1915 ha/year); the very different rates of change provide some perspective for this area in terms of drivers of land use change for this time period. The IOH in our study area has few secondary roads, (which have been correlated with increased rates of settlement deforestation [20]), and it is likely that rates in our study area are lower than for large expanding urban areas such as the city of Puerto Maldonado. However, above all else, the environmental and ecological effects of artisanal mining at this scale overshadow to a large degree, those typical of settlement deforestation [36].

Spatial patterns of gold mining are markedly different than that of settlement deforestation. While most mining areas follow streams and rivers, there are many small pockets of mining that are farther removed, yet necessarily have access to water for processing (i.e. wetlands or small streams). Interestingly, gold mining in this region seems to be occurring relatively independently of roads. For example, mining in Guacamayo (Figure 2A) began in late 2007 by river access to the north (>115 km upriver from the main city of Puerto Maldonado) and proceeded south towards the IOH. Colorado-Puquiri is accessed by either 250 km upriver travel or by the IOH followed by a major river crossing, and then on a road built after 2005 (at least two years after mining had begun).

Artisanal mercury-dependent gold mining in this region has occurred in the Andes since the time of the Inca [16], however it is now occurring at an entirely different scale. Very recently, mercury imports have increased exponentially (Figure 3) and reached an all-time high of ∼175 t in 2009. Virtually all Peru's imported mercury is used in artisanal mining [16]. We estimate mercury imports may be as high as 500 t for 2011, given a constant rate of increase in gold price.

Artisanal gold mining in Madre de Dios is occurring along waterways and wetlands guided by the presence of deposits and water bodies necessary for extraction. Mercury is being released directly into waterways and sediments, and is carried to biological channels through methylation and subsequent bioaccumulation and magnification. This area is a valuable headwater region for the Amazon River as an ecosystem service for the thousands of people along the river as well as the species that depend upon it [37]. Mercury released to the atmosphere during the artisanal heating of the gold-amalgam [7] eventually settles and then can be re-released through biomass burning [38], a common practice for clearing in settlement deforestation. In addition to the relatively well known human health and environmental risks, there is also evidence of a higher incidence of malaria infection with mercury exposure [39].

The effects of mercury have been relatively understudied or monitored in this region in recent years (but see [40]). Studies of mercury concentrations undoubtedly need to be continued and expanded in the physical and biological environment including humans and dispersion by methylation. With the latest innovations in publically available global high resolution topographic elevation models (30-m ASTER [41] and 90-m Shuttle Radar Topography Mission Data [42]), more complex spatial models of hydrology, biology and contaminant flow would be useful to predict the main sources and sinks for mercury through the watershed. Spatial predictive models of mining activity and gold deposits (human access, neighboring mines, geomorphology) could be useful in indicating areas that may be converted for mining in the near future. For many areas and commodities of the world, the increasing availability of satellite imagery time series [43] will hopefully motivate more studies that link economic variables with land use and cover changes on the ground.

In terms of policy approaches, Peru's newly created Ministry of Environment is actively struggling with the illegal mining issue [44]; a recent effort to reign in mining was made through a moratorium on new mining concessions. Peru has been recognized as having the potential to be a world leader in mercury stewardship [16]. Thus far national and regional-led efforts tend to focus on curtailing unapproved or illegal mining (e.g. using police forces, fines, or equipment seizures), strengthening the mining approval process, and improving mining practices to minimize mercury exposure. Control and regulation of mining will be difficult over the shorter term without national level restrictions on mercury imports and given the activity's economic importance [45], [46]. At the global level, a recently approved agreement to tackle mercury contamination by the United Nations Environmental Programme may support the Peruvian government's efforts [47]. Other alternatives such as fair trade gold (e.g. Alliance for Responsible Mining), technology innovations (e.g. [48]), and miner environmental education (e.g. [49]), will hopefully have an effect in the future, though major environmental improvements will not likely happen over the next few years.

Inasmuch as the data shown here applies to other similar mining operations in developing countries, the negative consequences to the environment and human health throughout the mining and drainage areas may ultimately prove to be catastrophic unless prompt control and remediation steps are taken. We predict that conditions will worsen with a stable or increasing price of gold.

## Supporting Information

Figure S1 Relationships among variables over time. A) International gold price and area deforested due to mining in areas of Madre de Dios, Peru. B) International gold price and Peruvian mercury imports 2002–2009.
(TIF)

Table S1 **Deforestation over time.**
(DOC)

Table S2 **Observed and predicted gold prices and mercury imports.**
(DOC)

Table S3 **Area of land conversion, 2009.**
(DOCX)
