# Supplementary material for: Gold Mining in the Peruvian Amazon: Global Prices, Deforestation, and Mercury Imports
Source: PLoS One. 2011 Apr 19;6(4):e18875. doi: 10.1371/journal.pone.0018875 (PMC3079740; doi:10.1371/journal.pone.0018875)
Supplement: Table S1 — Deforestation over time. (DOC) [file pone.0018875.s002.doc]

**Table S1. Deforestation over time.**

| **Geographic areas** | **Area deforested 2003, ha** | **Area deforested 2006, ha** | **Area deforested 2009, ha** | **Annual deforestation rate**  **2006-2009** |
| --- | --- | --- | --- | --- |
| Guacamayo | 0 | 0 | 1810.4 | 905.2* |
| Colorado-Puquiri | 431.3 | 1309.5 | 5244.4 | 1311.7 |
| Settlement deforestation (within 4 km of 100-km transect of IOH)** | --- | 12051.4 | 12723.7 | 224.1 |

Primary forest conversion by mining and settlement deforestation from 2003 to 2009. Primary forest removed by mining activities includes ponds, tailings, bare soil, standing dead trees, and small areas of heavily impacted vegetation between mining sites. Settlement deforestation includes recently cleared areas, sparsely vegetated areas, agriculture and human settlements.

*Calculated over 2 years; no mining was detected in this area until after Sept 2007. The estimates of mining area are conservative as we focused only on isolated known sites of gold mining, whereas gold mining is occurring in a multitude of smaller sites scattered along the area’s rivers and streams.

**Accuracy for the settlement deforestation map (forest vs. non forest) was found to be 93% for 2009 along 40 km of the IOH. Validation was made based upon an available high resolution image (CBERS-HRC 2.7 m resolution, p 181e, r114-5, acquired 23 September 2008). Accuracy was assessed by 87 random points across the area of image overlap of the 2009 deforestation map. Comparable imagery is not available for 2006. Areas that changed between 2008 and 2009 were excluded from the assessment.
