# Supplementary material for: Gold Mining in the Peruvian Amazon: Global Prices, Deforestation, and Mercury Imports
Source: PLoS One. 2011 Apr 19;6(4):e18875. doi: 10.1371/journal.pone.0018875 (PMC3079740; doi:10.1371/journal.pone.0018875)
Supplement: Table S2 — Observed and predicted gold prices and mercury imports. (DOC) [file pone.0018875.s003.doc]

**Table S2. Observed and predicted gold prices and mercury imports**

|  | **2003** | **2006** | **2009** | **2012**** |
| --- | --- | --- | --- | --- |
| Gold price* $US | 350 | 604 | 972 | 1580 |
| Annual increase in mercury imports (%) | 1 | 6 | 42 | 99 |
| Mercury imports, tn/yr | 94 | 85 | 165 | >1000 |

Increase in the rate of gold price and mercury imports over three-year increments between 2003 and 2009, and projections for 2012.

* Gold prices were observed to increase ~18% annually

**Mercury values were projected by fitting an exponential function to the measured points of the form y(gold price) = y_0_+ ae^bx^ (*r*^2^>0.92, *P<* 0.001).
