# Supplementary material for: Gold Mining in the Peruvian Amazon: Global Prices, Deforestation, and Mercury Imports
Source: PLoS One. 2011 Apr 19;6(4):e18875. doi: 10.1371/journal.pone.0018875 (PMC3079740; doi:10.1371/journal.pone.0018875)
Supplement: Table S3 — Area of land conversion, 2009. (DOCX) [file pone.0018875.s004.docx]

**Table S3**. **Area of land conversion, 2009.**

| **Land conversion** | **Area 2009, ha** | **% area of immediate subarea*** |
| --- | --- | --- |
| Mining: Guacamayo and Colorado-Puquiri | 7054 | 1.3 |
| Mining: Guacamayo, Colorado-Puquiri, and Huepetuhe | 15,500 | 2.8 |
| Settlement deforestation | 12,723 | 2.3 |

*a 46 x 120-km area encompassing all mining areas and IOH within the satellite image (547,400 ha).
